# Laying of Corner-Stone, Southern Medical College

**Published:** 1892-07

**Authors:** 


The laying of the corner stone of the new building of the So. thern Medical College took place with imposing ceremonies on the 28th of June. Dr. T. S. Powell, president of the board of trustees, and founder of the college, was the orator of the occasion. The new building will be ready for occupancy at the usual time for opening in October. It will accomodate over three hundred students in the amphitheatre, when it shall have been completed. The Southern Medical College is to be immediately in front of the Grady Hospital, and will derive great clinical advantage therefrom.

